# Diffusion of Information throughout the Host Interactome Reveals Gene Expression Variations in Network Proximity to Target Proteins of Hepatitis C Virus

**DOI:** 10.1371/journal.pone.0113660

**Published:** 2014-12-02

**Authors:** Ettore Mosca, Roberta Alfieri, Luciano Milanesi

**Affiliations:** Institute of Biomedical Technologies, National Research Council, Segrate, Milan, Italy; Saint Louis University, United States of America

## Abstract

Hepatitis C virus infection is one of the most common and chronic in the world, and hepatitis associated with HCV infection is a major risk factor for the development of cirrhosis and hepatocellular carcinoma (HCC). The rapidly growing number of viral-host and host protein-protein interactions is enabling more and more reliable network-based analyses of viral infection supported by omics data. The study of molecular interaction networks helps to elucidate the mechanistic pathways linking HCV molecular activities and the host response that modulates the stepwise hepatocarcinogenic process from preneoplastic lesions (cirrhosis and dysplasia) to HCC. Simulating the impact of HCV-host molecular interactions throughout the host protein-protein interaction (PPI) network, we ranked the host proteins in relation to their network proximity to viral targets. We observed that the set of proteins in the neighborhood of HCV targets in the host interactome is enriched in key players of the host response to HCV infection. In opposition to HCV targets, subnetworks of proteins in network proximity to HCV targets are significantly enriched in proteins reported as differentially expressed in preneoplastic and neoplastic liver samples by two independent studies. Using multi-objective optimization, we extracted subnetworks that are simultaneously “guilt-by-association” with HCV proteins and enriched in proteins differentially expressed. These subnetworks contain established, recently proposed and novel candidate proteins for the regulation of the mechanisms of liver cells response to chronic HCV infection.

## Introduction

Hepatitis C virus (HCV) infection is one of the most common chronic viral infections in the world and hepatocellular carcinoma (HCC) is the third-leading cause of cancer death worldwide. Chronic infection with HCV is the primary risk factor for developing HCC [1] but the sustained viral response in the treatment of HCV is associated with HCC reduction among treated population [2].

HCV is an enveloped, positive-stranded RNA virus belonging to the *Flaviviridae* family. Six major HCV genotypes and more than 100 subtypes have been identified. HCV is able to establish a chronic infection in 50–80% of exposed individuals and its infection largely follows a clinical course that after decades may result in liver fibrosis and cirrhosis in a subset of infected patients. HCV has a RNA genome of 9.6 kb translated into a unique polyprotein, which is subsequently processed by host and viral proteases into 10 proteins: three structural proteins, core, envelope (E) 1 and E2, and seven non-structural (NS) proteins p7, NS2, NS3, NS4A, NS4B, NS5A and NS5B. HCV proteins have been shown to interact with well-established cellular pathways, known to be involved in HCC initiation or progression: cell proliferation and differentiation involving epidermal growth factor (EGF) signaling pathway, Ras and Jak/STAT signaling pathway, PI3K-Akt pathway, wnt/-catenin signaling pathway, inflammation with NF-kB pathway, angiogenesis with the VEGF pathways, DNA damage response pathways with mitochondrial oxidative stress and ATM pathway [3]–[4]. Although the role of HCV in the onset of HCC is established, there is still the need for a systematic characterization of viral and host factors that can modulate the stepwise hepatocarcinogenic process from preneoplastic lesions (cirrhosis and dysplasia) to the neoplastic stages of HCC.

The increase of publicly available molecular interaction data, e.g. protein-protein interactions (PPIs), has enabled genome wide analyses of the activity of single units (e.g. gene expression studies) in the framework of the molecular interaction networks that regulate cell dynamics. Network-based approaches offer the possibility to address the analysis of biological systems taking into account that most of the biological functions arise from interactions among many components. Several studies have shown that network-based approaches lead to the identification of more robust markers and better stratifications of samples [5]–[6]. These approaches have also been used for studying the pathogenesis of HCV infection and its relation with HCC. For example, Drozdov *et al*. [7] defined a consensus gene relevance network for HCC progression; Zheng *et al*. [8] reconstructed stage-specific networks of PPIs enriched in differentially expressed genes during the progression from normal to HCV-induced HCC; He *et al*. [9] reconstructed stage-specific, deregulated networks of protein-protein and transcriptional regulatory interactions; Mukhopadhyay *et al*. [10] proposed infection gateway host proteins and possible pathways of HCV pathogenesis leading to various diseases.

However, it is still not clear whether acute and chronic effects of HCV activity can be explained according to a local impact hypothesis [11], i.e. in network proximity to host proteins targeted by viral proteins (HCV targets). Recently, using Epstein-Barr virus (EBV) and human papillomavirus (HPV), Gulbahce *et al*. (2012) have shown that host targets of viral proteins reside in network proximity to products of disease susceptibility genes and that the large proportion of the effect related to viral activity can be explained locally in intracellular networks [11]. In our study, we developed a computational approach to examine the relation between HCV targets, host protein-protein interaction (PPI) topology, pathways that regulate HCV response and the expression variations observed in samples collected from preneoplastic (cirrhotic, shortly “CIR”) and neoplastic (hepatocellular carcinoma, shortly “HCC”) liver lesions of HCV-infected patients of two independent studies.

In order to define the region of the host interactome where HCV proteins could determine the most relevant impact, we use network propagation [12], a technique that permits to establish a ranking among all the proteins of a PPI network in relation to their location relative to a subset of proteins. Network propagation can be seen as the diffusion of information from a subset of vertexes to all the others according to graph topology. Recently, network propagation has revealed its benefits in different problems, such as the association of genes and protein complexes with diseases [12], the stratification of tumor mutations [13], the identification of biomarkers in genome-wide studies [14], [15] and the relation between viral (EBV and HPV) perturbations and disease etiology [11]. We describe the use of network propagation for predicting the host proteins that are in a relevant position of the PPI network on the basis of HCV-host interactions and show that network propagation successfully prioritizes proteins that are involved in the host response to HCV. Subsequently, we show that networks of proteins “guilt-by-association” with HCV are significantly enriched in genes differentially expressed in cirrhotic (CIR) liver samples compared to normal (NORM) liver samples and in hepatocellular carcinoma (HCC) compared to CIR liver samples. These subnetworks contain established, recently proposed and novel candidate proteins for the regulation of the mechanisms of host response to acute and chronic HCV infection.

## Results and Discussion

### Viral-host and host protein-protein interaction data

We collected HCV-host PPIs from several systematic high-throughput screenings [16]–[18], the HCVPro database [19], the Host-Pathogen Interaction Database (HPIDB) [20], Intact [21] and VirHostNet [22]. These interactions were assessed through text mining and several experimental techniques, such as high-throughput yeast two-hybrid screens, 2-DE/Mass Spectrometry, affinity chromatography, coimmunoprecipitation, competition binding experiment, confocal microscopy, western blot, immunoblotting, metabolic labeling, mutational analysis, GST pull-down technique. We integrated the different datasets and obtained a list of 591 unique human proteins, shortly HCV targets, which interact with viral proteins.

We defined the human PPI network using only “high confidence” protein-protein pairs available in the STRING database [23]. A total of 517 HCV targets establish at least one PPI in the host interactome (Tab. S1).

### Identification of human proteins in network proximity to HCV targets

**Figure 1:**
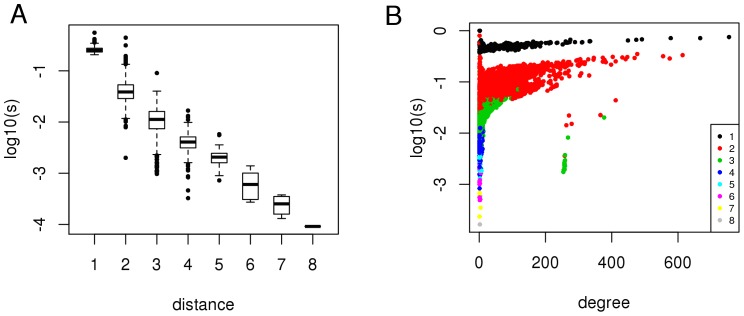
The network proximity score of a protein depends on network distance from viral targets and protein degree. **A**) Boxplots of network proximity scores (log_10_, vertical axis) grouped by viral-host protein network distance (horizontal axis). **B**) Network proximity scores (log_10_, vertical axis) distributed by protein degree (horizontal axis); colors indicate viral-host protein network distance.

We simulated the effects of HCV-host interactions throughout the molecular interaction network of the host cell to define the region of the host interactome that regulates the chain of events following the interaction between viral and host proteins. We considered the 517 HCV targets as causal proteins for studying the diffusion of the effects of HCV-host interactions, and used network propagation [12] to rank all the other proteins in relation to their network proximity to HCV targets in the PPI network. For each protein we obtained a score *s_i_* of network proximity to HCV targets: a short distance (number of links of the shortest path connecting two proteins) between a protein and any HCV target in the network (Fig. 1A) and a high number of interactions (degree) (Fig. 1B) are the two main factors that determine a high *s_i_*. Hence, network propagation gives a high rank to HCV targets and hubs (proteins with a high degree) of the PPI network (Tab. S1).

**Figure 2:**
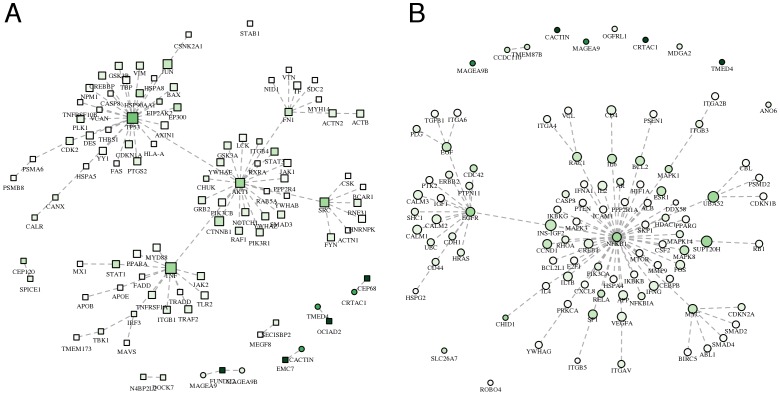
Most relevant relationships between proteins in network proximity to HCV proteins. MSTs among the (**A**) 100 proteins with the highest network proximity score (*s_i_*) and (**B**) 100 proteins with the highest *s_i_* excluding HCV targets, using edge weights (*w_ij_*) inversely proportional to the product of proteins network proximity scores: *w_ij_* = 1 - *s_i_s_j_*. **A–B**) The darker the color, the higher the network proximity score; squares: HCV targets; circles: non-HCV targets; vertex size is proportional to the number of interactions in the host interactome.

To summarize the results of network propagation in a meaningful and interpretable network representation, we used the so-called minimum spanning tree (MST), i.e. the tree that connects all the vertexes using the edges that determine the minimum sum of edge weights. Thus, by means of the definition of edge weights, it is possible to obtain MSTs that highlight different biological aspects. For example, defining edge weights as function of *s_i_*, the MST summarizes the relationships between proteins in network proximity to HCV targets. Note that, this type of MST clearly shows that hubs of the PPI network known to be relevant in HCV response (e.g. TP53, TNF, AKT1, SRC, FN1, NFKB1, MYC and EGFR) receive a high *s_i_* (Fig. 2, panels A and B).

In order to assess whether the scores of network proximity to HCV are specifically related to HCV targets, we repeated 1,000 times the network propagation procedure, each time using as source of information a different set of 517 proteins randomly sampled among all the host proteins. Therefore, we obtained 1,000 random network proximity scores for each protein. At this point, we calculated, for each protein, the probability *p_i_* of obtaining, by chance, a network proximity score higher than the one obtained using the 517 HCV targets as sources of information. If *p_i_* is low, then the *s_i_* of the corresponding protein is specifically related to HCV targets.

**Figure 3:**
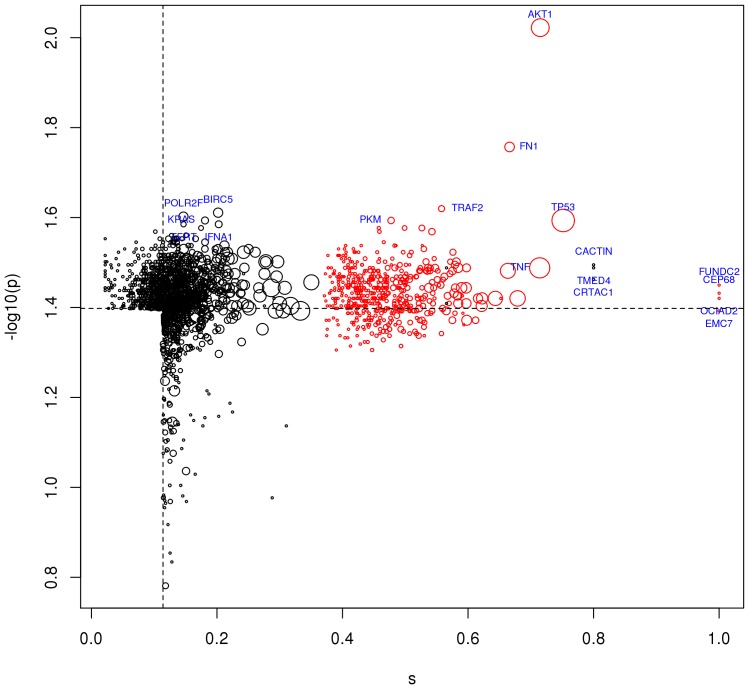
Top ranked proteins in network proximity to HCV targets. The top ranked 1,500 proteins by network proximity score *s_i_* (on the right of the dotted vertical line) or *p*-values (above the dotted horizontal line); red: HCV targets; black: non-HCV targets; point size is proportional to the number of interactions; labels indicate the top 10 of each ranking.

HCV targets that are hubs of the host interactome, like AKT1, TP53, TNF and FN1, received the most significant *p*-values (Fig. 3, red circles). Besides HCV targets, among the top ranked proteins, for example, we found BIRC5, KRAS, IFNA1 and TERT, which have well defined associations with HCV infection and propagation [24]–[27] (Fig. 3, black circles). Others, like CACTIN, TMED and CRTAC have not yet been clearly associated with HCV, but considering the significance of their network proximity to HCV targets these proteins may represent other players in the complex network that is involved in the response to HCV infection.

The two possible rankings, obtained by network proximity scores or *p*-values, have a strong overlap, which is maximal when considering the top 1,500 proteins of the two lists (Fig. S1). Note that proteins highly ranked according to network proximity scores are of biological interest despite their possible lower ranks when ordered by *p*-values, because high network proximity scores may indicate important gateways in the PPI network.

The biological significance of the proteins in network proximity to HCV has been further evaluated considering published lists of proteins that mediate host response to early and chronic HCV infection and HCC. The lists of proteins identified in this study show significant overlaps (*p*<<0.05) with HCC specific co-expression network [7], stage-specific differentially expressed networks in the progression of HCV induced HCC [8], proteins found in at least 20 papers related to HCV induced cirrhosis and HCC, and Hepatitis C associated genes according to the database DisGeNet [28] (Tab. 1, Tab. S2). Note that the significance of the overlaps is not affected by the exclusion of HCV targets, which occur in all the lists (Tab. 1). These overlaps suggest that key players of early and chronic processes that are induced by HCV infection lie in network proximity to HCV targets and can be predicted using currently available PPI data.

**Table 1:** Proteins in network proximity to HCV targets are highly enriched with lists of proteins proposed as regulators of host response to HCV and involved in HCC.

Source	Description	Ranking by			
		*p*	*s*	*p*	*s*
		with HCV targets		without HCV targets	
Drozdov *et al*. [7]	Consensus network for HCC	−6.96	−10.1	−5.13	−13.46
Zheng *et al*. [8]	Network of genes diff. expr. in CIR-NORM	−6.40	−7.86	−5.66	−7.10
Zheng *et al*. [8]	Network of genes diff. expr. in DYS-CIR	−7.93	−11.8	−11.4	−10.3
Zheng *et al*. [8]	Network of genes diff. expr. in eHCC-DYS	−9.29	−7.77	−8.92	−9.73
Zheng *et al*. [8]	Network of genes diff. expr. in aHCC-eHCC	−9.41	−8.67	−11.6	−14.2
ProteinQuest [65]	Genes associated with HCV and CIR	−7.97	−18.3	−8.28	−10.6
ProteinQuest [65]	Genes associated with HCV and HCC	−12.78	−18.2	−6.66	−7.67
DisgeNet [28]	Genes associated with Hepatitis C	−48.4	−75.2	−36.2	−52.3
DisgeNet [28]	Genes associated with chronic Hepatitis C	−48.4	−75.2	−36.2	−52.3

The table lists the log_10_ of the *p*-values that estimate the probability of obtaining, by chance (hypergeometric test), the observed overlap between the list of proteins from the literature (source and description, Tab. S2) and the top ranked 1,500 proteins in network proximity to HCV on the basis of *s_i_* or *p_i_*, including or excluding HCV targets; NORM  =  normal, CIR  =  cirrhosis, DYS  =  dysplasia, eHCC  =  early HCC, aHCC  =  advanced HCC.

### Transcriptional variation in preneoplastic and neoplastic liver samples

The transcriptional response in different phases of the HCV-dependent hepatic disease (cirrhosis and hepatocellular carcinoma) is an important aspect for joint investigation with the viral-host PPI network. To clearly understand biological mechanisms involved and altered during the different stages of hepatic disease, we focused on two gene expression datasets collected from the Gene Expression Omnibus (GEO) database [29] that are comparable in terms of histological characteristics (normal, cirrhotic and neoplastic tissues), viral infections (HCV) and microarray platform used. The dataset GSE6764 [30] includes 75 samples from cirrhotic and neoplastic livers of 38 HCV-infected patients and healthy livers of 10 patients. The dataset GSE14323 [31] includes 108 samples from cirrhotic, neoplastic and normal tissues from 88 HCV-infected patients and 19 HCV seronegative patients.

**Figure 4:**
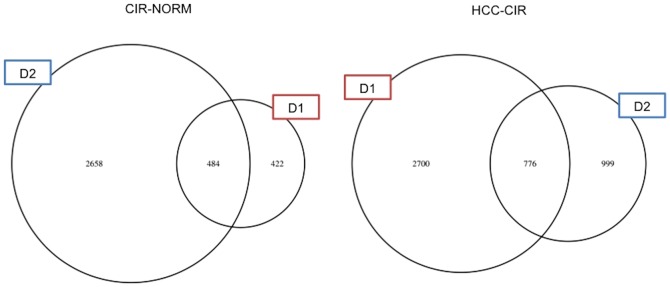
Venn diagram of the common DE genes for CIR-NORM and HCC-CIR contrasts. The number of DE genes is shown for each dataset (D1 and D2) for the two contrasts. The CIR-NORM and HCC-CIR contrasts show respectively 484 and 776 common DE genes between D1 and D2. The number of DE genes depends on the platform used for the microarray experiments.

To increase the reliability of gene expression variation in cirrhosis and HCC, we considered the differentially expressed (DE) genes in common between the two datasets as representatives for each state. In summary, the genes differentially expressed in the same direction (up- or down-regulation) in both the datasets considering CIR-NORM and HCC-CIR comparisons are respectively 484 and 776 (Fig. 4).

We evaluated the functional enrichment of the genes DE in both datasets to identify over-represented pathways (Tab. 2). The most abundant pathways found for the CIR-NORM contrast are related to the virus entrance into the host cell and the consequent signaling involved in both innate and adaptive inflammatory host defenses, as expected when an infection occurs [26], [32]. Moreover, we found the ECM-receptor interaction pathway, which is related to tissue and organ morphogenesis and associated with the maintenance of cell/tissue structure and function, as expected for the liver tissue going from normal to cirrhotic/fibrotic tissue. Nevertheless, already in this early phase we found one pathway involved in cancer (small lung cancer). The HCC-CIR contrast is mostly characterized by cancer-related pathways, with a significant number of genes involved in p53 signaling pathways and cell cycle. The cytokine-cytokine receptor interaction is a pathway highly represented in HCC-CIR showing that the innate/adaptive inflammatory host defenses is still on-going and that processes like cell growth, differentiation, angiogenesis are required for the neoplastic transformation.

**Table 2:** Functional enrichment of the genes differentially expressed in preneoplastic and neoplastic liver samples.

Contrast	Category	Term	Count	P-value	Benjamini
**CIR-NORM**	REACTOME	Signaling in Immune system	22	2.12E-04	0.00412
	REACTOME	Signaling by PDGF	10	1.62E-04	0.00632
	REACTOME	Integrin cell surface interactions	10	9.76E-04	0.0126
	KEGG	Focal adhesion	19	2.83E-04	0.0148
	KEGG	Cell adhesion molecules (CAMs)	15	2.45E-04	0.0192
	KEGG	ECM-receptor interaction	11	7.78E-04	0.0203
	KEGG	Antigen processing and presentation	11	7.07E-04	0.0221
	KEGG	Chemokine signalling pathway	17	0.00102	0.0228
	KEGG	Type I diabetes mellitus	8	6.46E-04	0.0252
	KEGG	Small cell lung cancer	12	1.82E-04	0.0284
	KEGG	Allograft rejection	7	0.00158	0.0308
	REACTOME	Axon guidance	7	0.00432	0.0413
	KEGG	Graft-versus-host disease	7	0.00243	0.0417
**HCC-CIR**	KEGG	p53 signalling pathway	16	8.76E-06	0.00152
	KEGG	Cytokine-cytokine receptor interaction	34	3.39E-05	0.00295
	KEGG	Cell cycle	20	1.50E-04	0.00868
	KEGG	Prostate cancer	14	0.00239	0.0801
	KEGG	Pathways in cancer	34	0.00219	0.0911

The functional enrichment has been performed for 484 and 776 DE genes respectively from CIR-NORM and HCC-CIR comparisons considering Reactome and KEGG pathways. The column “Count” reports the number of genes differentially expressed in the corresponding pathway; column “Benjamini” indicates the adjusted *p*-value.

### Expression variation in network proximity to HCV targets

In order to elucidate possible mechanistic relations linking HCV activity and the host response in terms of the gene expression variations observed in the subsequent liver lesions, we jointly analyzed human proteins for (i) the proximity to HCV targets and (ii) expression variation.

Initially, we assessed the significance of the overlap between the set of HCV targets and the sets of genes differentially expressed between normal, cirrhotic and HCC liver samples in the two studies of Wurmbach et *al*. [30] and Mas et *al*. [31]. We observed a small overlap, indicating that viral targets, as a whole, display a marginally significant differential expression in the considered pathological states (Tab. 3).

**Table 3:** Enrichment in HCV targets of differentially expressed genes in preneoplastic and neoplastic liver lesions.

Source	Comparison	*p* (GSEA)	*p* (hyper)
Wurmbach *et al*. [30]	CIR-NORM	0.129	0.0194
Wurmbach *et al*. [30]	HCC-CIR	0.117	0.0446
Mas *et al*. [31]	CIR-NORM	0.0310	0.000467
Mas *et al*. [31]	HCC-CIR	0.173	0.00233

*P*-values (*p*) were computed with Gene Set Enrichment Analysis (GSEA) and hypergeometric (hyper) test.

Hence, to study the gene expression variation in the local neighborhood of HCV targets, we extracted subnetworks of PPIs in network proximity to HCV targets and enriched in differentially expressed genes. Specifically, for each of the two comparisons (CIR-NORM and HCC-CIR), we used a search heuristic based on multi-objective optimization [33] in order to identify subnetworks of PPIs composed of proteins (i) with a high network propagation score and (ii) differentially expressed in both the studies of Wurmbach *et al*. (2007) [30] and Mas *et al*. (2009) [31]. Since we formulated the problem of finding PPI subnetworks as a multi-objective optimization problem with two criteria, we found a set of optimal solutions (i.e. PPI subnetworks) that collectively form (an approximation of) the so-called Pareto frontier of the problem [34], i.e. solutions that can not be improved simultaneously for all the objectives.

**Figure 5:**
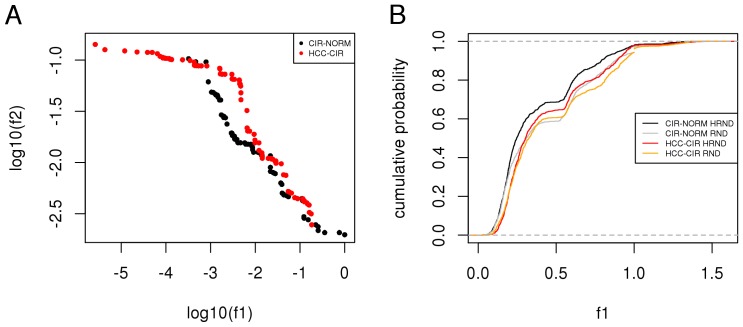
Differential expression in network proximity to HCV proteins. **A**) Gene expression variation (log_10_(*f*_1_)) and network proximity (log_10_(*f*_2_)) of optimal networks (Pareto fronts) identified for CIR-NORM and HCC-CIR comparisons. **B**) Estimated cumulative probability functions of gene expression variation (*f*_1_) of 1,000 random networks (RND) and 1,000 HCV associated random networks (HRND) in CIR-NORM and HCC-CIR comparisons. **A–B**) the lower the value of *f_i_* the higher the enrichment in the corresponding quantity.

In both the comparisons (CIR-NORM and HCC-CIR), we found optimal subnetworks with high network propagation score and enriched in differential expression (Fig. 5A). The Pareto front of CIR-NORM is composed of several points that dominate some of those of the HCC-CIR Pareto front. In other words, the multi-objective optimization procedure found subnetworks in network proximity to HCV targets and stronger differential expression in the CIR-NORM comparison.

To assess the significance of the relation between the neighborhood of HCV targets in the PPI network and differential expression, we generated 1,000 random subnetworks (shortly, RND subnetworks) and 1,000 random subnetworks in network proximity to HCV targets (shortly, HRND networks) (Fig. S2). We found that HRND subnetworks are significantly more enriched in expression variation than RND networks and, coherently with the results of multi-objective optimization, we found that HRND subnetworks have more significant differential expression in CIR-NORM compared to HCC-CIR (Fig. 5B and Tab. 4).

**Table 4:** Random subnetworks in network proximity to HCV targets are more enriched in differentially expressed genes than random subnetworks.

Comparison	*p* (KS) HRND vs RND	*p* (WMW) HRND vs RND
CIR-NORM	2.01E-05	4.11E-05
HCC-CIR	0.0388	0.0457

*p*-values were calculated using two-sample Kolmogorov-Smirnov (KS) and two sample Wilcoxon-Mann-Whitney (WMW) tests between the enrichment values (*f*_2_) for differential expression of 1,000 HRND and 1,000 RND subnetworks.

In order to visualize in a unique “summary” subnetwork all the optimal subnetworks extracted from the whole PPI network, for each comparison (CIR-NORM and HCC-CIR), we defined a summary subnetwork composed of all the proteins occurring in the relative optimal subnetworks. Then, we calculated the MSTs, in which each link (representing a PPI) was associated with a weight inversely proportional to the product of the absolute expression variation of the protein pair. Thus, these MSTs capture the PPIs between the most differentially expressed pairs of proteins in the neighborhood of HCV targets.

**Figure 6:**
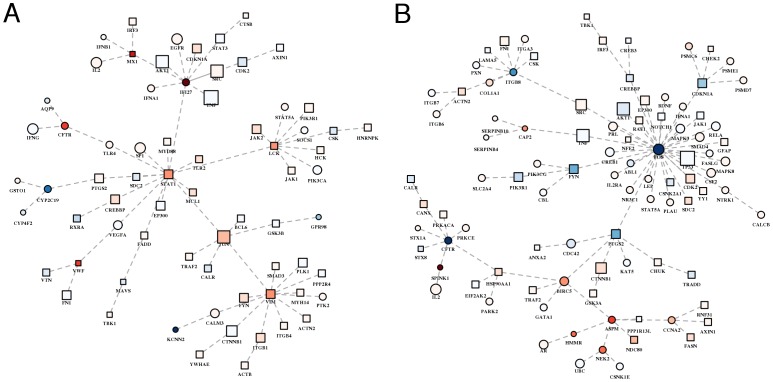
Most relevant relationships between proteins in network proximity to HCV and differentially expressed in preneoplastic and neoplastic liver samples. **A**) CIR-NORM. **B**) HCC-CIR. **A–B**) Colors, from blue (lower values) to red (higher values): average fold change; squares: HCV targets; circles: non-HCV targets.

The summary PPI network for the CIR-NORM comparison (Fig. 6A) has a higher network proximity to HCV targets than the CIR-NORM summary PPI network. The majority of the genes in the summary network of CIR-NORM regulate the immune system (45 out of 72, FDR  = 2.32E-13), the hemostasis (23/72, FDR  = 1.04E-8) and the cell-cell communication (10/72, FDR  = 1.57E-5), as expected from the available experimental evidences which suggest that HCV has direct and indirect roles in the pathogenesis of liver disease (Tab. S3). In fact, HCV is able to induce immunopathological effects and to promote liver disease, such as steatosis, fibrosis and cirrhosis [35]. Some hubs of the CIR-NORM subnetwork do not show significant variation in their expression (e.g. TNF, SRC, CDK2, AKT1) while others show significant up-regulation (STAT1, JUN, VIM). In particular, considering the nodes with a significant up- or down-regulation, we found that most of them are known to be involved in HCV-dependent pathways. For example, among the highly up-regulated genes, we found: MX1, which has an antiviral activity against a wide range of RNA viruses [36]; VWF, which is a new marker of liver fibrosis [37], IFI27, whose overexpression inhibits HCV replication and virus production [38]. Interestingly, we found one of the up-regulated genes, CFTR, which has not yet been associated with HCV infection but mainly associated to liver disease in cystic fibrosis [39]. The CIR-NORM network also involves highly down-regulated genes, such as CYP2C19, which is associated with a risk of HCC development [40] and KCNN2 involved in the trans-epithelial secretion in biliary epithelial cells and mainly expressed in normal liver [41].

The HCC-CIR subnetwork (Fig. 6B) is composed of proteins that are mainly involved in signalling by EGFR (17 out of 104, FDR  = 4.23E-6) and WNT (12/104, FDR  = 2.45E-3) in cancer, signalling by interleukins (13/104, FDR  = 1.22E-5), hemostasis (27/104, FDR  = 4.23E-6), apoptosis (12/104, FDR  = 4.97E-4) and cell cycle (19/104, FDR  = 6.66E-3). The results of the pathway analysis are compliant with several experimental evidences that identify the oncogenic role of the HCV proteins in the pathogenesis of HCC [42] (Tab. S4). In this network, we found some hubs included in the CIR-NORM network, such as TNF, TP53 and AKT1 with no significant expression variation. Concerning the up-regulated genes, we found: NEK2, also confirmed by Drozdov *et al*. (2012) [7]; ASPM, which is known as a molecular marker of hepatocellular carcinoma [43]; SPINK1, recently proposed as potential hepatocellular carcinoma marker [44]; HMMR, recently proposed as promoter of tumor metastasis [45]. Among the down-regulated genes we found FOS, an important regulator of tumor development [46], which is involved in a regulatory network together with JUN and SIRT6 [47]. We have also found CFTR, which is down-regulated in HCC-CIR.

### Progression of gene expression variation in HCV-mediated HCC

**Figure 7:**
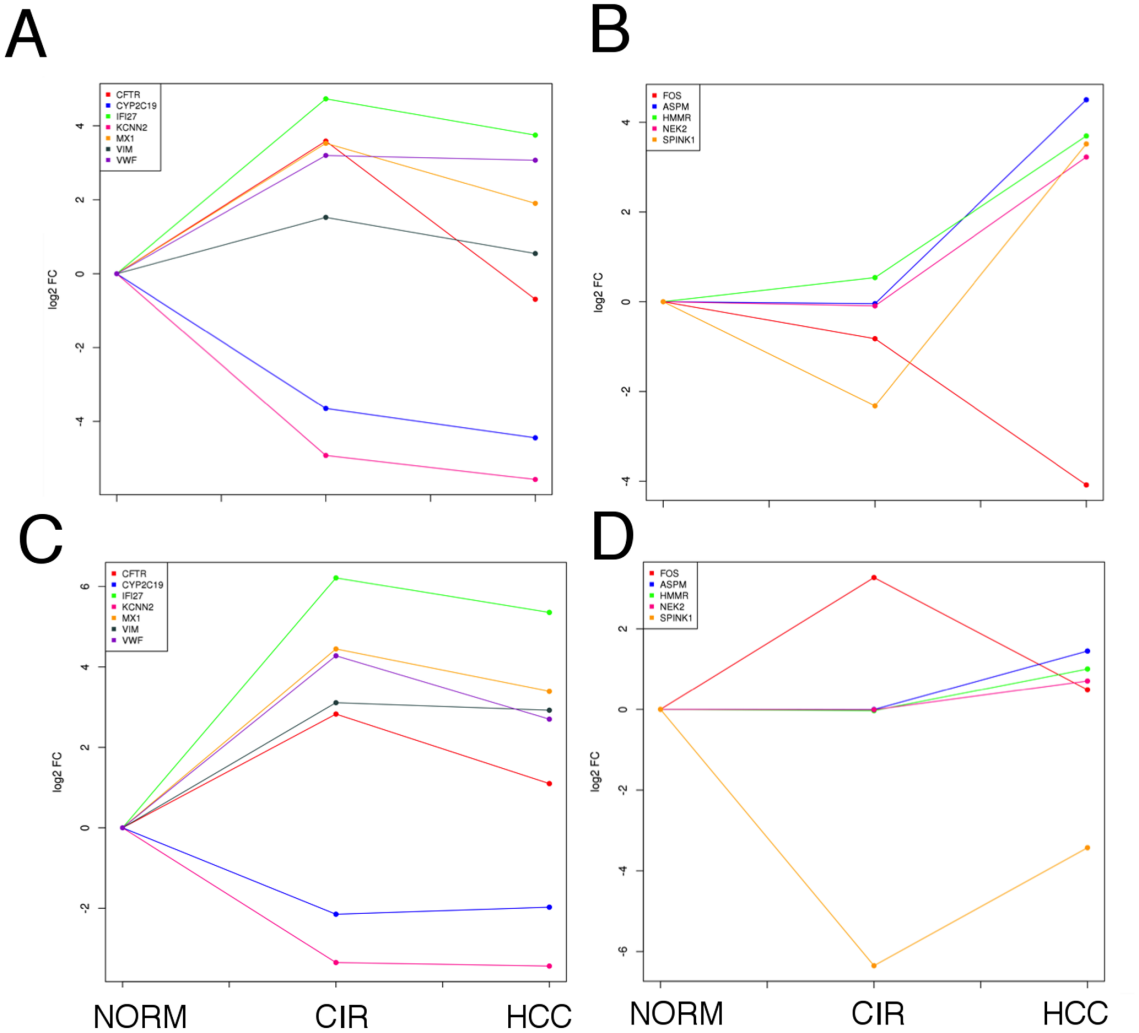
Expression variation in normal, cirrhotic and hepatocellular carcinoma samples of the most differentially expressed proteins that lie in network proximity to HCV targets. Mean log_2_ fold change (vertical axis) in NORM, CIR and HCC samples (horizontal axis). **A, C**: genes derived from the CIR-NORM summary PPI network (Fig. 5A). **B, D**: genes derived from the HCC-CIR summary PPI network (Fig. 5B).

We monitored the expression variation of the extremely up- or down-regulated genes found in the CIR-NORM and HCC-CIR summary PPI networks (absolute mean log_2_ fold change value greater than 2 in both datasets). In the early phase of the hepatic disease, five genes showed a significant up-regulation and two showed a significant down-regulation in both datasets (Fig. 7, panels A and C). IFI27 is the most up-regulated gene following the same trend in both datasets: very high increase between normal and cirrhosis followed by a small decrease between cirrhosis and HCC. The trend indicates that the expression of this gene was primarily affected at the disease onset, confirming its protective action against the early stages of HCV infection [48]. A similar trend is observed for VWF and MX1. Conversely, CFTR showed a significant up-regulation followed by a strong down-regulation in both studies. VIM, an hepatic stem cell marker [49], was found up-regulated in both datasets since the early phase of the hepatic disease. Concerning the down-regulated genes, we found that CYP2C19 expression is lower in cirrhosis samples than normal ones in both datasets, and remains low in HCC. This trend confirms that this gene plays an important role in the early stages of the disease and it is also associated with HCC development [50]. The other down-regulated gene is KCNN2, which follows the same trend as CYP2C19 and it remains down-regulated in cancer [51].

In the cirrhosis-hepatocellular carcinoma transition (HCC-CIR), we found three genes (HMMR, ASPM, NEK2) that do not show significant expression variations in the early phase of the disease progression (CIR-NORM), but are up-regulated in association with tumor progression, suggesting their specific relationship with tumor onset and development (Figure 7, panels B and D). The case of SPINK1 is peculiar: this gene was markedly down-regulated in cirrhotic compared to normal samples and then it is strongly up-regulated in HCC, confirming its potential role as a new marker for HCC, as recently proposed by Marshall *et al*. (2013) [44]. FOS displays a strong decrease in its expression level compared to the CIR-NORM condition.

## Conclusions

The list of PPI occurring inside human cells is a precious source of information to drive the interpretation of “omics” screenings [5], despite its limits [52], [53].

Simulating the impact of HCV-host interactions throughout the host PPI network, we have shown how proteins and pathways that are involved in HCV response and in the subsequent pathological states can be predicted considering HCV-host PPI interactions and the topology of the host PPI network. Our analysis provides evidences that, similarly to EBV and HPV [11], the effects of HCV-host interactions lie in network proximity to viral targets.

Beyond viral-host PPIs, our analysis has considered the transcriptional activity of liver cells collected from HCV-infected patients, in order to characterize the different phases of the hepatic disease. We observed that the differential expression detected in preneoplastic and neoplastic liver samples by two independent studies occur in network proximity to HCV targets, which conversely display, as a whole, weaker gene expression variations.

By simultaneously analyzing viral-host PPIs and gene expression variations observed in the early phase of the hepatic disease (CIR-NORM), and comparing hepatocellular carcinoma and cirrhotic samples (HCC-CIR), we extracted the subnetworks of interacting genes that summarize the expression variations observed in network proximity to viral targets. These subnetworks reveal the interactions between established, recently proposed and novel candidate proteins for the regulation of the mechanisms of liver cells response to chronic HCV infection. The expression variations observed in CIR-NORM occur in higher network proximity to viral targets than those observed in HCC-CIR. This difference is coherent with the view that cancer cells require the perturbation of other pathways in addition to those that are activated by the host as a consequence of HCV infection.

The computational approach presented in this work can also be applied for studying other biological processes that can be brought back to a finite set of causal biological entities.

Considering the growing interest in developing modulators of PPIs [54], [55], it will be important to design network-based approaches for the identification of “druggable” PPIs, selectively relevant for cells in pathological conditions. This will require a better understanding of the PPIs that are in fact occurring in the specific conditions of the living systems under analysis.

## Methods

### Protein-protein interaction data

We used the PPIs available in the STRING database v9.0 with score greater than 0.7, designated as “high confidence”. Multiple pairs of protein identifiers referring to the same pair of Entrez Gene identifiers were summarized according to the highest score. We obtained a PPI network composed of a total of 14,116 unique human proteins involved in 223,088 PPIs. We used the viral-host interactions collected from De Chassey *et al*. (2008) [16], Kwofie *et al*. (2011) [18], Dolan *et al*. (2013) [19], HPIDB [20], Intact [21], VirHostNet [22] and defined 591 unique human proteins that interact directly with HCV proteins, 517 of which appear in the PPI network.

### Network Propagation

We used network propagation [12] to smooth the HCV interaction information over the PPI network. This method is closely related to a random walk with restarts on a graph. Specifically, we used the iterative algorithm of Zhou *et al*. [56]:
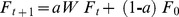
where F*_j_* is a vector of length *n* equal to the number of proteins of the PPI network, W is the *n*-by-*n* degree-normalized version of the adjacency matrix representing the PPI network, and *a* is a tuning parameter that establishes the relative importance of the two terms of the equation. This algorithm pumps the information available in the vertexes specified in F_0_ to their neighbors and, in turn, every vertex diffuses the information received during the previous iteration to its neighbors. The algorithm is run interactively for *t* = [0, 1, 2, …] until convergence: (F*_t_*_+1_- F*_t_*) <1e-6. The elements of W are obtained dividing the adjacency matrix A by the square root of the product of its row sums: *w_ij_* =  *a_ij_* (*d_i_ d_j_*)^-½^.

In our study, the 517 elements of F_0_ corresponding to HCV targets were initialized with value equal to 1, while all the other elements were set to 0. The parameter *a* was set to 0.8, a value that determined consistent results in previous studies [12], [13].

### Gene expression data analysis

The raw data of the two datasets were separately processed and analyzed using the statistical software R. The data normalization was carried out using the gcrma method available in the simpleaffy package [57]. Differential expression was assessed with the limma package [58]. We considered as differentially expressed the genes with *p*-value <0.05. We excluded two samples from the dataset GSE6764 because of quality issues, as reported by the authors [30]. We assessed the quality metrics of the microarray datasets using the arrayQualityMetrics package [59]. This quality control analysis did not identify any outlier.

### Significance of protein lists overlaps and gene set enrichment analysis

The statistical significance of the overlap between each pair of protein lists was calculated using the hypergeometric distribution implemented in R functions “phyper” and “dhyper”. The GSEA [60] of HCV targets and differentially expressed genes was carried out using the R package HTSanalyzeR [61] ranking the genes by their π-values in descending order. The π-value is a recently proposed statistic that takes into account both the fold change and *p*-value [62]: the higher the π-value, the more significant the difference between the two samples.

### Random subnetworks generation

Random subnetworks were created by random extension from randomly chosen “seed” proteins. For each subnetwork, while the number of protein was less than 10, a seed was selected and a maximum of 3 of its neighbors were added. After the first iteration, the seed protein was randomly selected among current nodes. In order to create 1,000 random (RND) subnetworks and 1,000 random subnetworks in network proximity to HCV targets (HRND) we used two different pools of seeds: 1,000 proteins tossed among all the human proteins (RND subnetworks) and the 1,000 proteins with the highest network propagation score (HRND subnetworks). This procedure ensured the definition of two sets of subnetworks with significantly different network proximity to HCV targets (Fig. S2).

### Functional Annotation

The functional enrichment analysis to identify over-represented KEGG [63] and Reactome [64] pathways in DE gene lists was carried out using the Database for Annotation, Visualization and Integrated Discovery (DAVID) [65]. We considered the adjusted *p*-values provided by DAVID in the functional annotation chart under the name “Benjamini”. The pathway analysis of the two summary subnetworks for CIR-NORM and HCC-CIR was carried out using the over-representation analysis tool provided by Reactome [66]. This analysis determined which events (pathways or reactions) were statistically enriched in the two summary subnetworks (Tab. S5, S6).

### Text Mining

Literature-based text mining was performed using ProteinQuest (PQ) [67]. PQ is a web based platform for biomedical literature retrieval and analysis. PQ searches within PubMed abstracts and extracts the text of the image captions from free full text articles. PQ text-mining tool parses target documents searching for terms related to curated ontologies (e.g. diseases, bioprocesses, pathways, body parts). Multiple searches for more than one alias were used to resolve ambiguities in the terminology. We considered the following queries: “HCV AND CIR”, “HCV AND HCC”. Then, we calculated the number of co-occurrences of two terms (query and protein) in a minimum of 20 papers among those retrieved by each query. We obtained 71 and 75 proteins respectively for HCV-CIR and HCV-HCC queries.

### Multi-Objective optimization

The search of PPIs subnetworks in (i) network proximity to HCV targets and (ii) differentially expressed was formulated as the multi-objective optimization problem of minimizing two objective functions. We solved this problem using an evolutionary algorithm that creates a population of subnetworks extracted from the whole PPI network and, then, iteration by iteration, modifies the subnetworks (adding and removing vertexes) in order to minimize simultaneously the objective functions [33]. Given *x*, a subset of the proteins included in the PPI network that form a connected subnetwork, *s*, the list of network propagation scores ranked in descending order, *e*_D1_ and *e*_D2_, the lists of expression variations (log_2_ fold changes) in two datasets D1 and D2, ranked in descending order, *x*_up_ and *x*_down_, the subsets of proteins of *x* that are, respectively, up-regulated and down-regulated in both datasets D1 and D2, we defined:
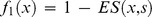





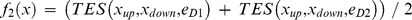



The quantity ES(*x*, *y*) is the enrichment score [60], it assumes values in the real interval [−1,1] and indicates to which extent the elements of the set *x* are located at the top (ES ->1) or at the bottom (ES ->−1) of the ranked list *y*. The quantity TES(*x*, *z*, *y*)  = 1 - (ES(*x*, *y*) - ES(*z*, *y*))/2 is the inverse total enrichment score [68]; it assumes values in the real interval [0, 2] and it tends to 2 when the elements of *x* occur at the top of the ranked list *y*, while the elements of *z* occur at the bottom of *y*. Therefore *f*_1_ will be low if the subnetwork *x* is enriched in proteins in network proximity to HCV targets, while *f*_2_ will be low if the subnetwork *x* is enriched in proteins differentially expressed (up-regulated and down-regulated) in both datasets D1 and D2.

We used the gene expression datasets from Wurmbach *et al*. [30] and Mas *et al*. [31]. We ranked gene expression differences on the basis of π-values [62] multiplied by the sign of the corresponding log fold change, in order to obtain up-regulated genes at the top and down-regulated genes at the bottom of the ranked list.

For each comparison, CIR-NORM and HCC-CIR, we run 10 times the multi-objective optimization using a population of 500 subnetworks (ranging from a minimum of 10 to a maximum of 50 vertexes) for 1,000 iterations. Subsequently, we defined the optimal subnetworks as those belonging to the non-dominated set (Pareto front), considering all the subnetworks generated for each comparison.

## Supporting Information

Figure S1 **Similarity between the rankings of host proteins obtained using network proximity scores or** ***p*** **-values.** The similarity between the two ordered lists *x* and *y* was calculated as the mean of the enrichment score (ES) of the top of the list *x* in the list *y* and the ES of the top of the list *y* in the list *x*: sim  = 1/2 * (ES(*x*_top_, *y*) + ES(*y*_top_, *x*)). We varied the definition of the tops ranging from 100 to 5,000 elements and observed the highest similarity when considering the top 1,500 elements of the lists. The similarity observed using several random lists of the same lengths is definitely lower.
(TIF)

Figure S2 **HCV-associated random networks.** Estimated cumulative probability functions of HCV association (*f*_2_, the lower the value the higher the association) of 1,000 random networks (RND) and 1,000 HCV-associated random networks (HRND).
(TIF)

Table S1 **Top ranked proteins according to the network proximity to HCV targets.** Columns - “is HCV target”: 1 (yes), 0 (no); “score”: network proximity score scaled in the [0, 1] interval; “score p-value”: estimated probability of obtaining *s_i_* by chance; “degree”: number of PPI.
(XLS)

Table S2 **Published lists of proteins that mediate host response to early and chronic HCV infection and HCC.**
(XLS)

Table S3 **Reactome pathway analysis of the CIR-NORM summary subnetwork.** List of the statistically enriched pathways in the CIR-NORM subnetwork resulting from Reactome over-representation analysis.
(XLS)

Table S4 **Reactome pathway analysis of the HCC-CIR summary subnetwork.** List of the statistically enriched pathways in the HCC-CIR subnetwork resulting from Reactome over-representation analysis.
(XLS)
