# Supplementary figures and images for: Diffusion of Information throughout the Host Interactome Reveals Gene Expression Variations in Network Proximity to Target Proteins of Hepatitis C Virus
Source: PLoS One. 2014 Dec 2;9(12):e113660. doi: 10.1371/journal.pone.0113660 (PMC4251971; doi:10.1371/journal.pone.0113660)

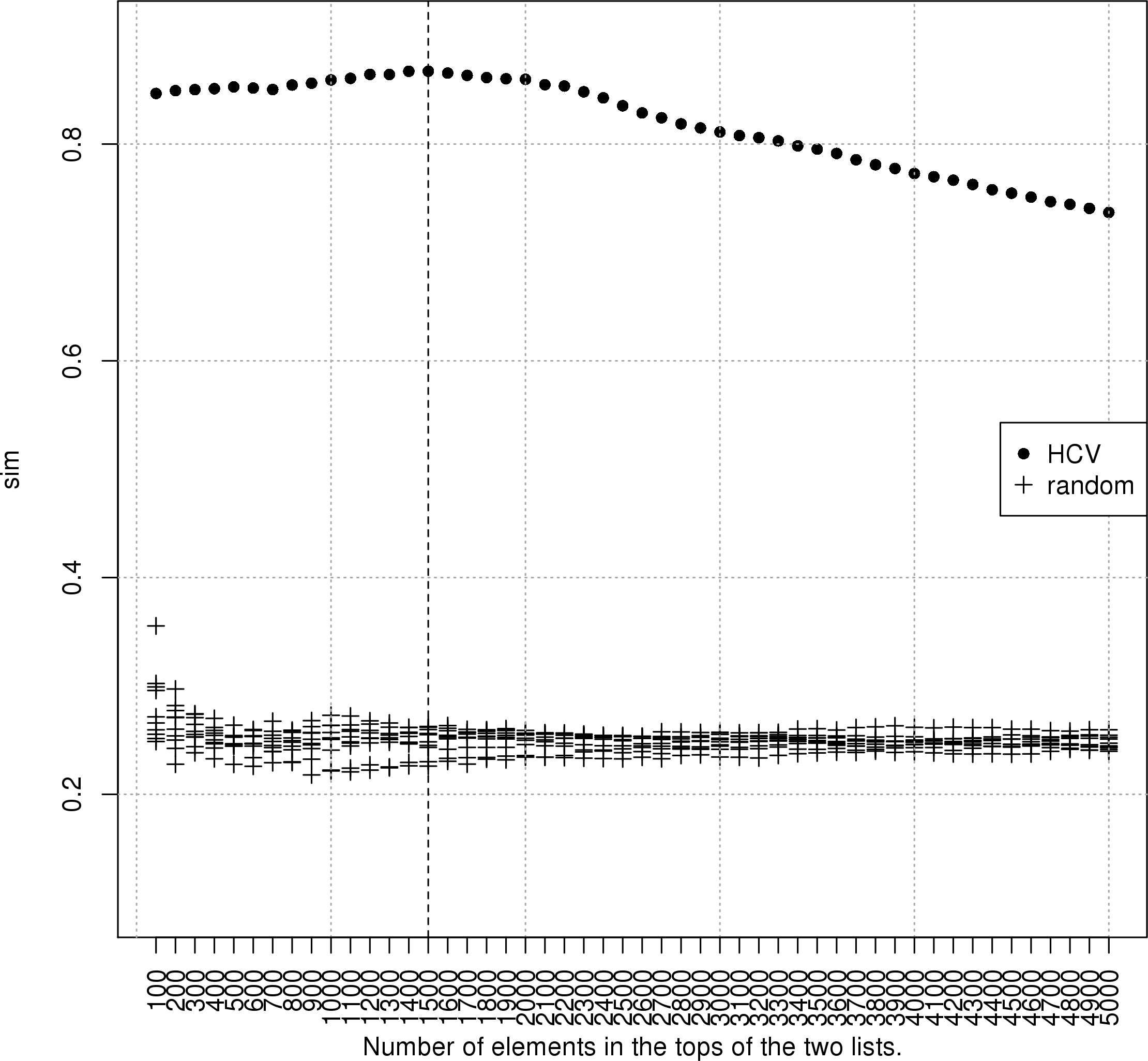

Supplement: Figure S1 — Similarity between the rankings of host proteins obtained using network proximity scores or p -values. The similarity between the two ordered lists x and y was calculated as the mean of the enrichment score (ES) of the top of the list x in the list y and the ES of the top of the list y in the list x: sim = 1/2 * (ES(x top, y) + ES(y top, x)). We varied the definition of the tops ranging from 100 to 5,000 elements and observed the highest similarity when considering the top 1,500 elements of the lists. The similarity observed using several random lists of the same lengths is definitely lower. (TIF) [file pone.0113660.s001.tif]

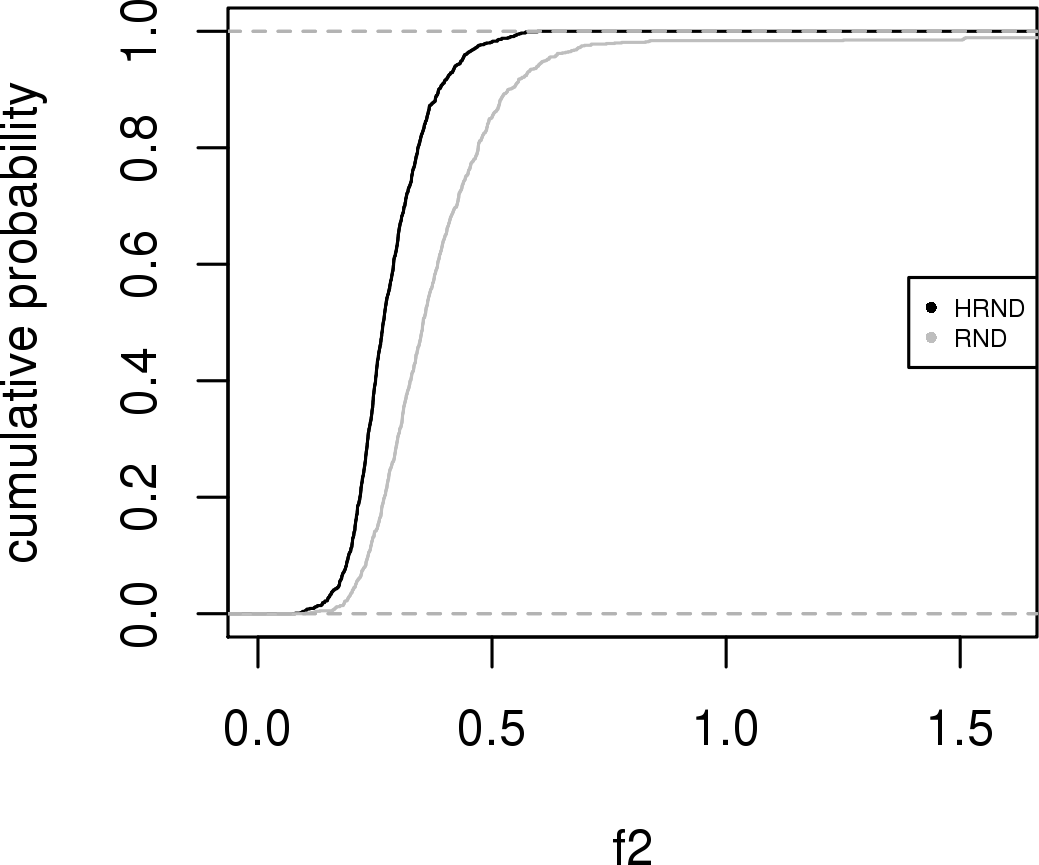

Supplement: Figure S2 — HCV-associated random networks. Estimated cumulative probability functions of HCV association (f 2, the lower the value the higher the association) of 1,000 random networks (RND) and 1,000 HCV-associated random networks (HRND). (TIF) [file pone.0113660.s002.tif]
